# Osteogenesis Imperfecta in neonatal period in Cameroon: A case report

**DOI:** 10.1002/ccr3.3572

**Published:** 2020-11-20

**Authors:** Ritha Carole Mbono Betoko, Suzanne Ngo Um Sap, Marie‐Ange Ngo Yamben, Jocelyn Tony Nengom, Paul Koki Ndombo

**Affiliations:** ^1^ Université Of Douala Faculty of Medicine and Pharmaceutical Sciences Douala Cameroon; ^2^ University of Yaounde, Faculty of Medicine and Biomedical Sciences Yaounde Cameroon; ^3^ Mother and Child Centre Chantal Biya Foundation Yaounde Cameroon

**Keywords:** bone fragility, fractures, neonatal period, Osteogenesis Imperfecta

## Abstract

Early forms of Osteogenesis Imperfecta should be considered as main etiology of bone deformities in newborns. Prenatal diagnosis and genetic counseling should be improved in Africa. Management of these children remains difficult in low‐income countries.

## Graphical abstract

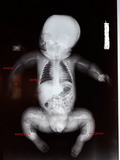

## 1 INTRODUCTION

Osteogenesis Imperfecta is a genetic disorder of connective tissue leading to bone fragility. Diagnosis of this condition can be considered during pregnancy, at birth or during childhood. Early‐onset forms are severe with high mortality rate during pregnancy or the first hours of life. We report two cases of neonatal diagnosis of this condition in Cameroon.

Osteogenesis Imperfecta (OI) is a group of inherited disorders of connective tissue caused by mutations in one of the two genes encoding for type 1 collagen.^1^ Clinical features include bone fragility and low bone mass resulting in bone fractures, bone deformity, and growth impairment. This disorder affects approximately 0.3‐0.7/10.000 births.^2^ Age at diagnosis varies depending on the OI type. The *Sillence* classification reported four types depending on clinical, radiological, and genetic features.^3^ This classification was extended by some authors to include new genetic forms of OI due to recessive inherited mutations.^4^ Types II and III are the most severe forms with high mortality rate during antenatal and neonatal period. In sub‐Saharan Africa, few cases of neonatal diagnosis have been described.^5^ We report two cases of neonatal diagnosis of this rare condition in Cameroon.

## 2 CASE PRESENTATION

### 2.1 Case 1

**Figure 1:**
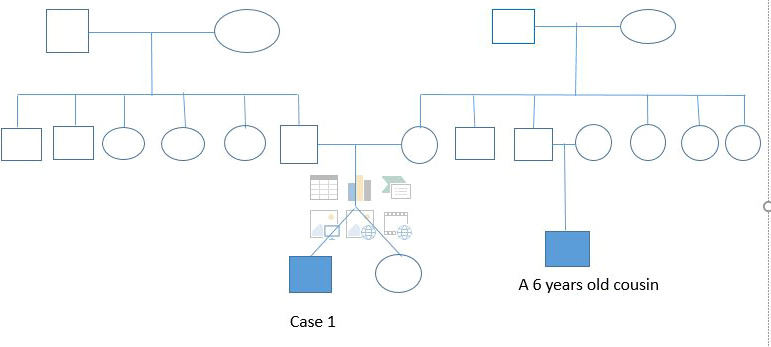
Case 1 Pedigree

We received a 26‐day‐old male newborn who was referred from a regional hospital for multiple fractures and limb deformities discovered at birth. He was born at 39 weeks through vaginal route with breech presentation and a birth weight of 2700 g. It was an uneventful twin pregnancy. Antenatal laboratory tests were normal. Three ultrasounds were done during prenatal period with no reported abnormalities. There was no consanguinity, no family history of short stature. Limb deformity was found in a 6‐year‐old cousin but the underlying cause was unclear (Figure 1). The twin sister was in good health at birth.

**Figure 2:**
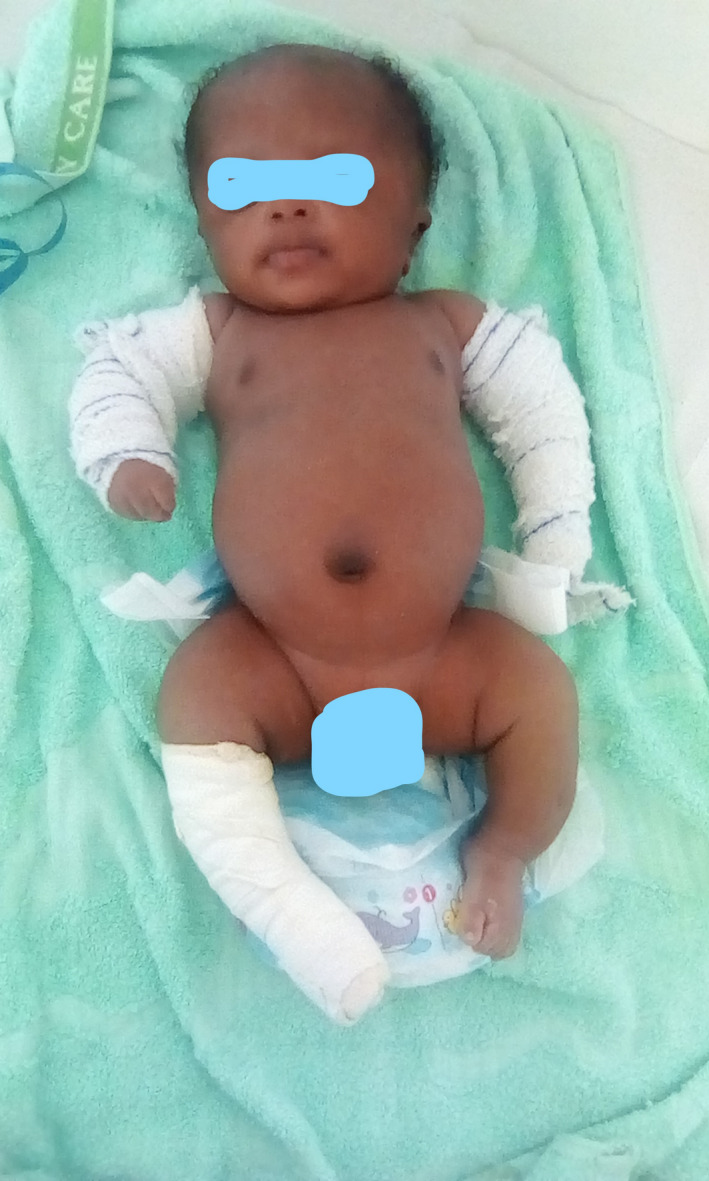
Bowed and shortened limbs

**Figure 3:**
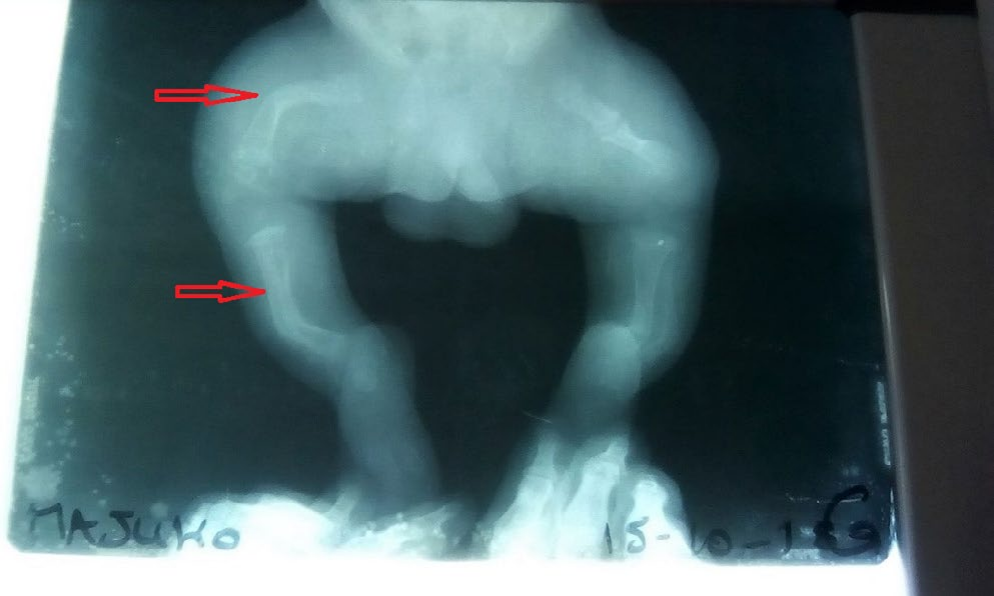
Multiple fractures of bones

At clinical presentation, anthropometric parameters included a weight of 3200 g (−1 SDS), height 47 cm (−2 SDS), and head circumference of 35 cm (+ 0.5 SDS). The child presented with reduced mobility, frontal bossing, white sclerae, moderate respiratory distress, bowed legs, and shortened limbs (Figure 2). Limbs X‐rays showed multiple diaphyseal fractures of long bones, demineralization, and curved bones (Figure 3). Transfontanellar brain ultrasound and cardiac ultrasound were normal. Laboratory findings included normal calcium (97.2 mg/L) and increased alkaline phosphatases (374.68 IU/L). Genetic tests are not yet available in our country. According to clinical and radiographic findings, Osteogenesis Imperfecta was the more likely diagnosis of this bone fragility. We suggested OI type III as diagnosis due to early presentation. For management of this condition, the child received orthopedic treatment for recent fractures and oral Vitamin D supplements. The child is currently 9 months old with a weight of 7750 g (−1 SDS), a height of 57 cm (‐ 3.5 SDS), and a head circumference of 44 cm. He recently received the first dose of bisphosphonates. A recent lumbar spine X‐ray revealed tiered vertebral collapse from T12 to L3 with no spinal deformities. The physiotherapist tried to maintain a semi‐sitting position to avoid spinal deformities.

### 2.2 Case 2

We received a 37‐day‐old male baby who was referred by a surgeon for medical management of multiple fractures and limb deformities discovered at birth. It was an eventful pregnancy with normal prenatal tests. A 3rd trimester ultrasound revealed femoral bowing and shortening. He was delivered through cesarean section at 37 weeks due to uterine scar from a 32‐year‐old mother. He had a birth weight of 3000 g and a height of 40 cm. At birth, he presented with limb deformities and painful mobility of lower limbs. He was admitted for an early neonatal sepsis at day 2 of life with good improvement. He is the second child of the family with no family history of consanguinity nor limb deformities.

**Figure 4:**
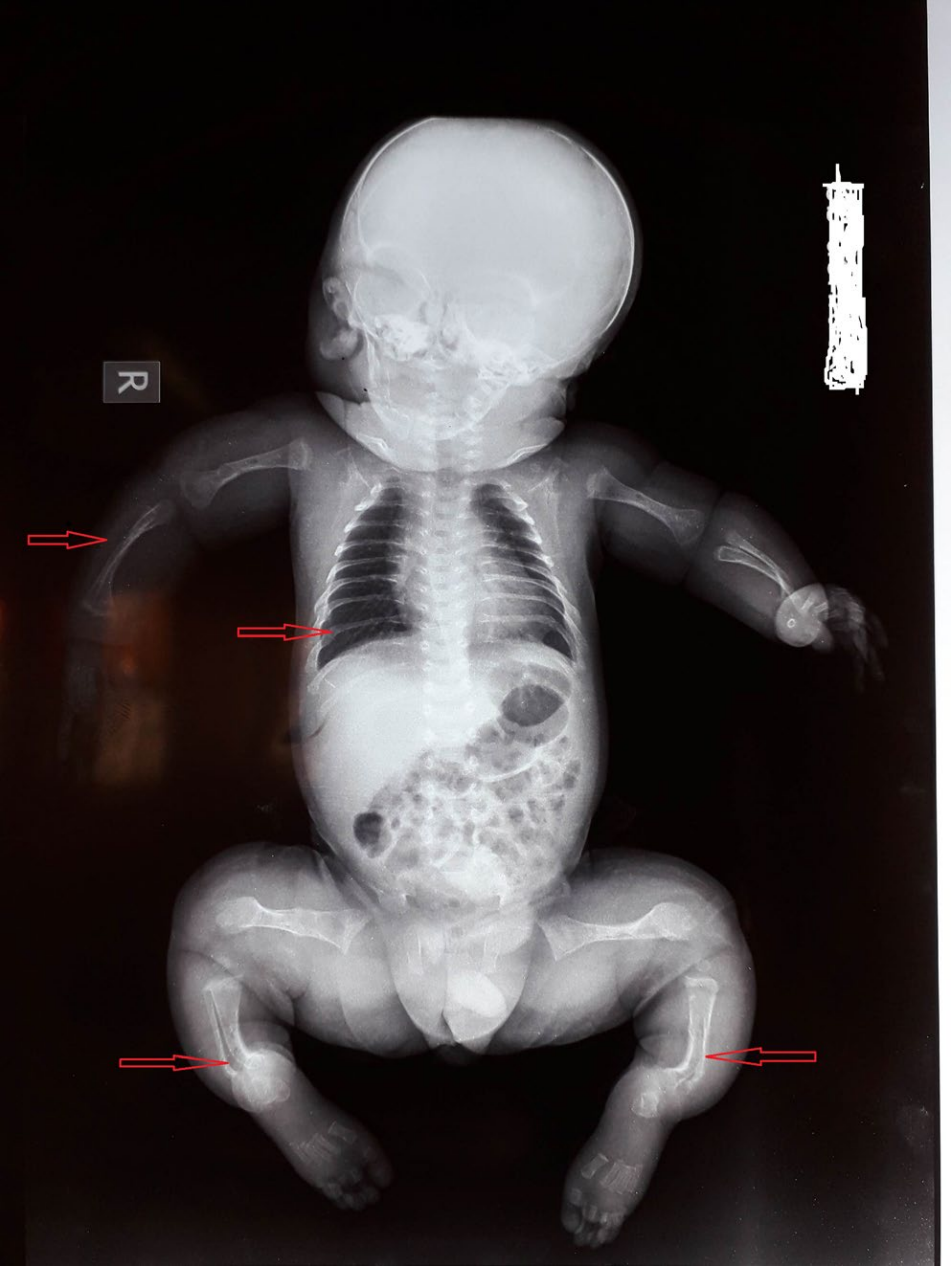
Incomplete fractures of long and curved bones

At clinical presentation, anthropometric measurements included a weight of 4000 g (−1 SDS), height of 45 cm (−2 SDS), and head circumference of 35 cm (+0.5 SDS). The child presented with reduced mobility, frontal bossing, blue sclerae, bowed legs, and shortened limbs. Radiography of the limbs showed multiple diaphyseal fractures of long bones and curved bones (Figure 4). The patient also presented with an undisplaced sternal fracture. Genetic tests are not yet available in our country. According to clinical and radiographic findings, Osteogenesis Imperfecta was the more likely diagnosis of this bone fragility. We suggested OI type II or III as diagnosis due to antenatal deformities and multiple fractures at birth. For management of this condition, the child received orthopedic treatment with several casts for recent fractures. Bone callous was formed after 8 weeks with pain improvement. He also received oral Vitamin D supplements. Bisphosphonates are planned but not yet available.

## 3 DISCUSSION

Osteogenesis Imperfecta is a common cause of bone fragility in children. Clinical diagnosis is usually based on skeletal signs as for our patients. This genetic disorder can also present with extraskeletal signs such as respiratory distress, cardiovascular abnormalities, dentinogenesis Imperfecta, blue sclerae, hearing impairment, and vascular fragility.^6^, ^7^ Genetic tests can confirm the diagnosis but molecular abnormalities of type 1 collagen (*COL1A1 and COL1A2*) are only reported in 85% of patients.^2^ Antenatal or neonatal onset of this condition as in our patient suggests a severe form with high mortality rate. These forms can be suspected on antenatal ultrasound (bone deformity, fracture, and bone shortening).^6^ These features were reported during antenatal ultrasound of our second patient. This highlights the importance of ultrasound for antenatal diagnosis of this condition.

*Sillence* classification distinguishes four types of OI based on clinical phenotypes and disease severity (Table 1).^3^ In 2004, Rauch et al added 3 other types to the *Sillence* classification..^4^ Types II and III are the most severe forms with antenatal or neonatal onset. Our patients probably had Type III OI due to early onset of bone fractures and deformities. Since 2006, other defects were discovered in genes encoding for proteins which are important for type 1 collagen metabolism. These new classifications describe previously known phenotypes but specific gene defects not related to collagen mutations (Table 2).^2^ The prognosis is guarded in neonatal forms due to intracranial hemorrhage or respiratory insufficiency following chest deformity.^6^ Respiratory distress of the first patient at admission caused fear of respiratory insufficiency or associated cardiovascular malformation. A normal cardiac ultrasound ruled out these comorbidities that are life‐threatening in severe forms. In addition, transfontanellar ultrasound eliminated intracranial hemorrhage.

**Table 1:** Sillence classification of Osteogenesis Imperfecta ^1^, ^2^

Type	Severity	Features	Inheritance
Type I	Mild	Blue sclerae, near normal stature, mild bone fragility, late‐onset hearing loss, no dentinogenesis imperfecta	Autosomal dominant
Type II	Lethal form	Blue sclerae, multiple intrauterine fractures, severe deformity, stillbirth or perinatal death	Autosomal dominant or recessive
Type III	Severely deforming	Normal sclerae, dentinogenesis imperfecta, short stature, scoliosis, frequent fractures	Autosomal recessive
Type IV	Moderately deforming	White sclerae, moderate bone fragility, short stature, possible dentinogenesis imperfecta,	Autosomal dominant or recessive

**Table 2:** Genetic classification of Osteogenesis Imperfecta ^3^

Type	Mutated gene	Encoded protein	Clinical features	Inheritance
Impairment of collagen synthesis and structure				
I, II, III or IV	COL1A1 or COL1A2	Collagen α1(I) (COL1A1) or α2(I) (COL1A2)	As described in Table 1	Autosomal dominant
Compromised bone mineralization				
V	*IFITM5*	Bone‐restricted interferon‐induced transmembrane protein‐like protein	Normal‐to‐severe skeletal deformity, normal‐to‐blue sclerae; hearing loss	Autosomal dominant
VI	*SERPINF1*	Pigment epithelium‐derived factor (PEDF)	Moderate‐to‐severe skeletal deformity, childhood onset	Autosomal recessive
Abnormal collagen post‐translational modification				
VII	*CRTAP*	Cartilage‐associated protein (CRTAP)	Severe rhizomelia with white sclerae	Autosomal recessive
VIII	*P3H1*	Prolyl 3‐hydroxylase 1 (P3H1)		Autosomal recessive
IX	*PPIB*	Peptidyl‐prolyl *cis*–*trans* isomerase B		Autosomal recessive
Compromised collagen processing and crosslinking				
X	*SERPINH1*	Serpin H1	Severe skeletal deformity, blue sclerae, dentinogenesis imperfecta, skin abnormalities, and inguinal hernia	Autosomal recessive
XI	*FKBP10*	65 kDa FK506‐binding protein (FKBP65)	Mild‐to‐severe skeletal deformity, normal‐to‐grey sclerae, and congenital contractures	Autosomal recessive
No type	*PLOD2*	Lysyl hydroxylase 2 (LH2)	Moderate‐to‐severe skeletal deformities and progressive joint contractures	Autosomal recessive
XII	*BMP1*	Bone morphogenetic protein 1 (BMP1)	Mild‐to‐severe skeletal deformity and umbilical hernia	Autosomal recessive
Altered osteoblast differentiation and function				
XIII	*SP7*	Transcription factor SP7 (also known as osterix)	Severe skeletal deformity with delayed tooth eruption and facial hypoplasia	Autosomal recessive
XIV	*TMEM38B*	Trimeric intracellular cation channel type B (TRIC‐B; also known as TM38B)	Severe bone deformity with normal‐to‐blue sclerae	Autosomal recessive
XV	WNT1	Proto‐oncogene Wnt‐1 (WNT1)	Severe skeletal abnormalities, white sclerae, and possible neurological defects	Autosomal dominant or recessive
XVI	*CREB3L1*	Old astrocyte specifically induced substance (OASIS; also known as CR3L1)	Severe bone deformities	Autosomal recessive
XVII	SPARC	SPARC (also known as osteonectin)	Progressive severe bone fragility	Autosomal recessive
XVIII	*MBTPS2*	Membrane‐bound transcription factor site‐2 protease (S2P)	Moderate‐to‐severe skeletal deformity, light blue sclerae, scoliosis, and pectoral deformities	X‐linked recessive

The management of OI is multidisciplinary including surgical and medical treatment of bone fragility.^8^ Orthopedic management or surgical treatment of fractures and bone deformities is essential during follow‐up.^6^ Physiotherapy helps to maintain mobility, maintain muscle mass, and improve motor skills. Bisphosphonates are considered as first‐line medical treatment for OI.^9^ Benefits of this treatment include reduced pain, increased bone mass density, and reduced incidence of fractures.^10^ The psychosocial support of families is necessary. In our country, management of these patients remains difficult due to low socio‐economic level.

## 4 CONCLUSION

Osteogenesis Imperfecta is a rare inherited disease leading to abnormal bone fragility. Early forms are reported to be severe. Therefore, caregivers should be aware of this condition when bone deformities and fractures are observed in a neonate. Genetic counseling and prenatal diagnosis should be considered in these families.

## CONFLICT OF INTEREST

None declared.

## AUTHOR CONTRIBUTIONS

All the authors managed these patients, searched literature, and revised the manuscript. MBRC and MANNY designed the manuscript.

## ETHICS STATEMENT

The parents of these children gave their consent for publication.

## Data Availability

Not applicable.

## References

[1] Forlino A , Marini JC . Osteogenesis imperfecta. Lancet Lond Engl. 2016;387(10028):1657‐1671. 10.1016/S0140-6736(15)00728-X PMC7384887 26542481

[2] Marini JC , Forlino A , Bächinger HP , et al. Osteogenesis imperfecta. Nat Rev Dis Primer. 2017;3:17052. 10.1038/nrdp.2017.52 28820180

[3] Sillence DO , Senn A , Danks DM . Genetic heterogeneity in osteogenesis imperfecta. J Med Genet. 1979;16(2):101‐116. 458828 10.1136/jmg.16.2.101 PMC1012733

[4] Rauch F , Glorieux FH . Osteogenesis imperfecta. Lancet Lond Engl. 2004;363(9418):1377‐1385. 10.1016/S0140-6736(04)16051-0 15110498

[5] Kaboré A , Cissé A , Yonaba C , et al. Ostéogenèse imparfaite : à propos de quatre cas à Ouagadougou (Burkina Faso). Pan Afr Med J. 2015;22 69 26834922 10.11604/pamj.2015.22.69.6299 PMC4725658

[6] Antoniazzi F , Mottes M , Fraschini P , Brunelli PC , Tatò L . Osteogenesis Imperfecta. Paediatr Drugs. 2000;2(6):465‐488. 11127846 10.2165/00128072-200002060-00005

[7] Lee DY , Cho T‐J , Choi IH , et al. Clinical and Radiological Manifestations of Osteogenesis Imperfecta Type V. J Korean Med Sci. 2006;21(4):709‐714. 16891817 10.3346/jkms.2006.21.4.709 PMC2729895

[8] Harrington J , Sochett E , Howard A . Update on the evaluation and treatment of osteogenesis imperfecta. Pediatr Clin North Am. 2014;61(6):1243‐1257. 25439022 10.1016/j.pcl.2014.08.010

[9] Dwan K , Phillipi CA , Steiner RD , Basel D . Bisphosphonate therapy for osteogenesis imperfecta. Cochrane Database Syst Rev. 2016;10:CD005088. 27760454 10.1002/14651858.CD005088.pub4 PMC6611487

[10] Garganta MD , Jaser SS , Lazow MA , et al. Cyclic bisphosphonate therapy reduces pain and improves physical functioning in children with osteogenesis imperfecta. BMC Musculoskelet Disord. 2018 19():344 10.1186/s12891-018-2252-y 30249227 PMC6154399

